# COVID-19 vaccination pharmacovigilance in Khojaly district, Uzbekistan: an epidemiological evaluation

**DOI:** 10.3389/fpubh.2025.1520821

**Published:** 2025-07-02

**Authors:** Yunis Tursinov, Roberta Horth, Botirjon Kurbonov, Alfiya Denebayeva, Shalkar Adambekov, Dilyara Nabirova

**Affiliations:** ^1^Central Asia Field Epidemiology Training Program, Almaty, Kazakhstan; ^2^Department of Epidemiology with the course of HIV infection, Asfendiyarov Kazakh National Medical University, Almaty, Kazakhstan; ^3^Department of the Republic of Karakalpakstan, Sanitary and Epidemiological Welfare and Public Health Service, Khojaly District Branch, Nukus, Uzbekistan; ^4^U.S. Centers for Disease Control and Prevention, Central Asia Office, Almaty, Kazakhstan; ^5^Sanitary and Epidemiological Welfare and Public Health Service of the Republic of Uzbekistan, Tashkent, Uzbekistan; ^6^Department of Epidemiology, Biostatistics, and Evidence-Based Medicine, Al-Farabi Kazakh National University, Almaty, Kazakhstan

**Keywords:** vaccines, adverse events following immunization, post-vaccine complications, COVID-19, Uzbekistan

## Abstract

**Introduction:**

COVID-19 vaccines are safe and effective, reducing global morbidity and mortality. Strong vaccine safety surveillance systems increase public confidence in vaccines. Regular evaluations are needed to ensure these systems function effectively. We evaluated the adverse events following immunization (AEFI) surveillance system for COVID-19 vaccine in Khojaly District, Uzbekistan.

**Methods:**

We analyzed National Vaccine Registry data for April 2021–March 2022 for Khojaly (population: 120,000). The registry captures demographic information for individuals who received or refused COVID-19 vaccination and reported AEFI. An AEFI is any untoward medical occurrence that follows immunization, but does not necessarily have a causal relationship with the vaccine. In June 2022, we also surveyed 30 consenting vaccination providers from five outpatient clinics and reviewed regulatory documents related to COVID-19 vaccination and AEFI reporting. We performed descriptive statistics using R.

**Results:**

A total of 78,453 COVID-19 vaccines doses were administered in Khojaly from April 2021 to March 2022. Of these, 70% were Zifivax (ZF2001), an adjuvant protein vaccine produced in Uzbekistan, 9% were Pfizer–BioNTech, 7% were Moderna, and 14% were others. There were 843 AEFI correctly reported by providers (1,074 per 100,000) during this time, 837 (1,067 per 100,000 doses) of which were reported as mild allergic reactions, 4 (5 per 100,000 doses) as exacerbations of chronic disease, and 2 (3 per 100,000 doses) as anaphylactic shock. Among the providers surveyed (*n* = 30), 15 (50%) had previously encountered at least one AEFI following COVID-19 vaccination, and 3 (10%) had encountered a severe AEFI. Among all providers, only 13 (43%) submitted an AEFI report, and 10 (33%) did not know where to report an AEFI. The most common barriers to reporting included having a large patient load (20%) and not having access to computers (7%). The AEFI surveillance system lacked a feedback loop to share summary data with healthcare facilities and clinicians for informed decision-making.

**Conclusion:**

The COVID-19 vaccination surveillance system in Khojaly recorded AEFIs, but we identified gaps in AEFI reporting and knowledge among providers. Improving the AEFI registry and training providers on standard operating procedures for identifying, reporting, and investigating AEFI could improve vaccine safety surveillance.

## Introduction

Vaccines against COVID-19 are safe and effective, and the introduction of vaccines has resulted in decreased morbidity and mortality from COVID-19 worldwide. By June 2023, Uzbekistan had more than 253,000 confirmed COVID-19 cases and 1,637 COVID-19 deaths (1). In March 2021, the first COVID-19 vaccines were approved for use in Uzbekistan (2), and within 2 years, at least 80 million vaccine doses were administered (1). Several COVID-19 vaccines were developed and released at an advanced pace as part of the emergency response to the pandemic. Over 18 million people (53% of the population) were vaccinated against COVID-19 in the country at least once during this period (3, 4). A domestically manufactured vaccine, Zifivax (ZF2001, trade name RBD-Dimer), was the most frequently used COVID-19 vaccine, accounting for approximately 60% of all doses. Zifivax is a recombinant COVID-19 vaccine developed in China by Anhui Zhifei Longcom in collaboration with the Institute of Microbiology of the Chinese Academy of Sciences. Clinical trials have demonstrated the high safety and efficacy profile of this vaccine (5).

Pharmacovigilance systems are critical for ensuring the safety of vaccines. Robust surveillance of adverse events following immunization (AEFI) is important for rapidly identifying and addressing safety signals that could affect individual and population health (6). An AEFI is globally defined as “any untoward medical occurrence that follows immunization, and that does not necessarily have a causal relationship to the vaccine” (7, 8). AEFI are considered severe if they are life-threatening, lead to hospitalization, permanent disability, congenital anomaly, congenital disability, or death, or require urgent medical care (7, 8). Mild AEFI are minor unfavorable or unintended signs, abnormal laboratory findings, and symptoms or diseases that resolve on their own and require limited medical intervention (9, 10). AEFI can be detected through passive and active surveillance. The World Health Organization (WHO) recommends reporting: (1) serious AEFI; (2) events associated with a newly introduced vaccine; (3) immunization errors that may have caused AEFI; (4) significant events occurring within 30 days after immunization; and (5) events causing significant concern from parents or the community. Capturing all events post-vaccination increases the sensitivity of identifying adverse events following immunization (AEFI). In Uzbekistan, healthcare providers are obligated to recognize, record, and report AEFI, and providers involved in vaccine administration are provided trainings on the correct diagnosis and reporting of AEFI (9).

**Figure 1:**
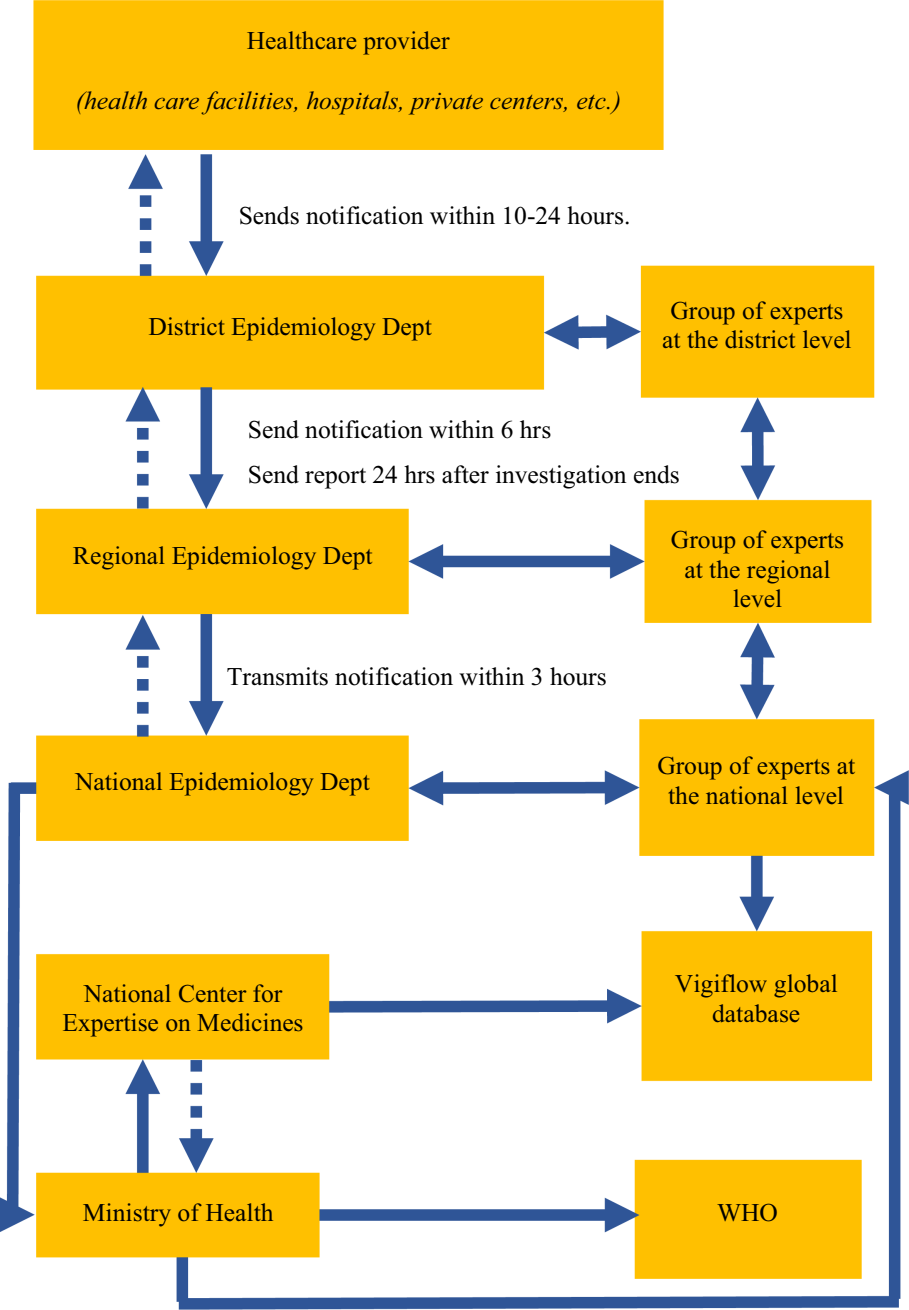
Diagram of AEFI surveillance in Uzbekistan.

Vaccine safety surveillance in Uzbekistan falls under the responsibility of the Sanitary and Epidemiological Welfare and Public Health Service, which is located within the Ministry of Health at the district, oblast, and national levels (Figure 1) (3, 11). The surveillance system for AEFI was first established in 2001. It is a passive surveillance system in which immunization staff and medical providers report any AEFI encounters by phone. The AEFI system in Uzbekistan does not capture direct reports from the population. In January 2021, an electronic system was developed to track the delivery of COVID-19 vaccines and all AEFIs, following COVID-19 vaccination, were reported into this system. The introduction of the electronic system brought certain adjustments, but did not replace the standard AEFI registration process by phone.

The large number of new COVID-19 vaccines being introduced in a short period of time during the COVID-19 pandemic requires particular attention to the safety and surveillance system of AEFI. A robust AEFI surveillance system in Uzbekistan, functional at different levels, is important for the rapid roll-out of vaccines during epidemics, including the SARS-CoV-2 pandemic. This study aimed to (1) document and describe AEFI; (2) evaluate the quality of AEFI data and understand knowledge of AEFI reporting through an evaluation of the COVID-19 vaccination surveillance system in the Khojaly district of Karakalpakstan.

## Methods

### Evaluation framework

We conducted a surveillance system evaluation using the U.S. Centers for Disease Control and Prevention’s Guidelines for Evaluation Public Health Surveillance Systems (12), and examined the quality (data completeness and validity) of the AEFI surveillance data for COVID-19 vaccines, the simplicity of its structure and operation, and the usefulness of the surveillance system. The evaluation took place between March and May 2022 in the Khojaly district of Uzbekistan, which has a population of 120,000.

### Data sources and collection

The district has 10 primary care clinics responsible for administering COVID-19 vaccines and reporting AEFI. We selected 5 clinics using convenience sampling based on the largest catchment population and geographic distribution (central and remote areas). In these five clinics, we extracted data from electronic and paper medical records, including outpatient records (13). We reviewed weekly and monthly AEFI reporting forms, and also obtained data from the national COVID-19 vaccine registry for 2021–2022 (14). We also interviewed 30 healthcare providers from the 5 selected clinics in the district. Providers were systematically selected from a list of all registered and available clinic staff responsible for vaccination and AEFI reporting. Specifically, every 5th provider was chosen from the list after a random starting point, with up to 6 providers selected per clinics.

We used interviewer-administered pretested questionnaire to obtain information about AEFI identification, detection, reporting, and registration (Appendix I). We piloted the questionnaire with 5 healthcare workers from a clinic that was not included in the study.

### Data analysis

The CDC evaluation of the existing AEFI surveillance system uses mixed methods (qualitative and quantitative), and no inferential analyses were performed. The analyses included descriptive statistics such as frequencies, percentages, incidence rates, and assessment of data completeness and validity. The qualitative method is the interviews of providers. Data were analyzed using R statistical software, version 4.1.0.

### Ethical information

Permission to conduct the study was obtained from the Ministry of Health of Uzbekistan. Verbal informed consent was obtained from all study participants, and participation in the study was voluntary. No personal identifying data were collected. This activity was reviewed by the U.S. Centers for Disease Control (CDC), deemed not research, and was conducted consistent with applicable federal law and CDC policy (45C. F.R. part 46.102(I)(2), 21C. F.R. part 56; 42 U.S.C. 241(d); 5 U.S.C. 552a; 44 U.S.C. 3,501 et seq.).

## Results

### AEFI system description and gaps

AEFI reporting is mandatory for all vaccines administered in the country. When COVID-19 vaccines were rolled out in Uzbekistan, a telephone-based notification system for AEFI reporting was introduced, and an electronic national registry was developed to capture patient vaccination data, including AEFI. AEFI were reported telephonically directly by healthcare facilities to the district epidemiology department (Figure 1). The district epidemiology department also called facilities daily, requesting AEFI case reports. District departments then reported to regional epidemiology departments within 24 hours, and regional departments reported to the national epidemiology department within 3 hours. All data were aggregated at the Ministry of Health and the National Center for Expertise on Medicines, which maintains the registry and conducts AEFI investigations.

The evaluation of AEFI surveillance system revealed several data quality concerns. Because AEFI reports at the health facility level were only completed over the phone, there were no written AEFI individual case reports retained at the health facility level for review. Facilities retained only weekly and monthly aggregate AEFI reports. Individual-level AEFI records were, therefore, only available electronically in the national COVID-19 vaccine registry.

A review of data in the electronic national COVID-19 vaccine registry revealed several data quality concerns. The first was related to the open text variable format of many variables. These variables could be entered in any language (Uzbek or Russian) and in Cyrillic or alphanumeric characters. This may have resulted in names being duplicated when they were entered in different ways. In addition, the registry did not allow for the entry of data for patients who refused the second and subsequent doses of the vaccine. Another limitation of the electronic system was that after the data were entered into the electronic registry, health departments could not edit the data. Therefore, when there were updates for people, a new entry had to be created. Moreover, the system had limited capabilities for analyzing or reviewing data for specific periods, and it was impossible for healthcare facilities to see AEFI at the healthcare facility level; these data were not shared back to healthcare facilities.

### AEFI reports

From April 2021 to March 2022, a total of 78,453 COVID-19 vaccines were administered in Khojaly, Karakalpakstan. Of these, 70% were Zifivax (ZF2001)^*^, 9% were Pfizer-BioNTech, 7% were Moderna, and 14% were other vaccines (Table 1). The National Vaccination Registry contained 3,444 records of AEFI. Of these, the majority, 2,601 (75%), were not true AEFI because healthcare workers incorrectly classified patients as having an AEFI if they refused the 2nd or 3rd dose of their COVID-19 vaccine regimen. The remaining 843 valid AEFI represented 1.1% of administered doses (1,074 per 100,000) during this time. Among these, 837 (1,067 per 100,000) were reported as mild injection site reactions, 4 (5 per 100,000) as exacerbations of chronic disease, and 2 (3 per 100,000) as anaphylactic shock (Table 2).

**Table 1:** COVID-19 vaccine doses and reported adverse effects following immunization (AEFI) by vaccine type in Khojaly district, Uzbekistan, 2021–2022.

Vaccines	Vaccinated^1^	Reported AEFI		
		Mild injection site reaction^2^	Exacerbation of chronic disease^2^*	Anaphylactic shock^2^
All COVID-19 vaccines	78,453 (100%)	837 (1.1%)	4 (0.005%)	2 (0.003%)
Zifivax (ZF2001)**	54,917 (70.0%)	526 (1.0%)	2 (0.004%)	1 (0.002%)
Pfizer-BioNTech	7,315 (9.3%)	33 (0.5%)		
Moderna	5,750 (7.3%)	163 (2.8%)		1 (0.017%)
Oxford/AstraZeneca (AZD1222)	4,803 (6.1%)	62 (1.3%)		
CoronaVac (Sinovac)	3,593 (4.6%)	26 (0.7%)	1 (0.028%)	
Sputnik V (Gam-COVID-Vac)	1903 (2.4%)	25 (1.3%)	1 (0.053%)	
Sputnik Light***	172 (0.2%)	2 (1.2%)		

^1^Column percent.

^2^Row percent.

^*^Diabetes; tuberculosis.

^**^Trade name RBD-Dimer, a recombinant COVID-19 vaccine developed by Anhui Zhifei Longcom in collaboration with the Institute of Microbiology of the Chinese Academy of Sciences.

^***^Single dose of Sputnik V (Gam-COVID-Vac).

**Table 2:** Reported adverse effects following immunization (AEFI) in Khojaly district, Uzbekistan, 2021–2022.

AEFI	*N*	%
Reported AEFI after COVID-19 vaccination	3,444	4.4%
Not AEFI (incorrectly reported)*	2,601	75.1%
Mild injection site reaction	837	24.3%
Exacerbation of chronic disease**	4	0.12%
Anaphylactic shock	2	0.06%

^*^An error in the electronic database due to the incorrect registration of the patient’s vaccine refusal.

^**^Diabetes; tuberculosis.

Mild injection site reactions were reported most frequently in people who received Moderna (2.8%) and least in people who received Pfizer-BioNTech (0.5%) (Table 1). Severe events such as the exacerbation of chronic diseases and anaphylactic shock were rare, occurring in fewer than 0.06% of recipients across all vaccine types.

There was no formal recording, reporting, or investigation of serious AEFI to determine if these were correct reports of true AEFI observed after administration of COVID-19 vaccines.

We reviewed individual patient medical charts for randomly selected patients with AEFI and found that most medical records were missing documentation from any medical examination conducted prior to vaccination. Outpatient cards, which are stored separately from patient medical records, were missing information required for AEFI reports, including body temperature, description of the injection site, and general health status of the patient. There was also no documentation about whether patients were followed up after 3 days postvaccination.

### Healthcare provider interviews

Half of the 30 providers interviewed reported having at least one patient with an AEFI observed after administration of the COVID-19 vaccine, and 43% submitted an AEFI report (Table 3). However, 33% of the providers did not know where to send AEFI reports, 23% could not correctly identify AEFI types, and only 20% considered weekly AEFI reporting important. When asked how long it took to register a case of AEFI, 8 (27%) providers responded that 5 min was enough, 8 (27%) thought 10 min was enough, 2 (6%) thought >10 min was needed to report AEFI, 8 (27%) providers said they did not know, and the remaining 4 (13%) gave various answers ranging from 1 hour to 1 day.

**Table 3:** Vaccine provider knowledge of adverse effects following immunization (AEFI) cases and reporting: Khojaly district, Uzbekistan, 2021–22.

Among vaccine providers	*N* = 30	%
Had a patient with an AEFI	15	50%
With a nonsevere AEFI (*N* = 15)	12	80%
With a severe AEFI (*N* = 15)	3	20%
Did not register an AEFI (*N* = 15)	2	13%
Submitted an AEFI report	13	43%
Did not know where to submit AEFI report	10	33%
Could not correctly identify types of AEFI	7	23%
Agree weekly AEFI reports are important	6	20%
Barriers to reporting an AEFI included:		
Large patient load	6	20%
Lack of computers and printed AEFI paper forms	2	7%
Limited number of staff to record in registers	1	3%
Lack of transportation for delivery of AEFI forms	1	3%
Insufficient support with this task	1	3%
The AEFI paperwork is burdensome	1	3%

Among the healthcare workers who had patients with AEFI, 80% had patients with mild AEFI, 20% had patients with severe AEFI, and 13% did not report an AEFI (Table 3). Among the 30 healthcare workers, 20% replied that they do not have time to manage the entire attached population and to follow up with patients for 3 days after vaccination, whereas 7% of employees answered that they have technical barriers to reporting and registration, such as an insufficient number of computers or reporting forms.

## Discussion

Our study evaluated the quality, simplicity of its structure, and usefulness of the AEFI surveillance system for COVID-19 vaccines in the Khojaly district of Karakalpakstan, Uzbekistan. It revealed that the AEFI surveillance system in Uzbekistan was useful and able to communicate AEFI quickly during the swift rollout of COVID-19 vaccines. However, we identified gaps in the reporting of AEFI, a lack of documentation in outpatient medical records, and important gaps in healthcare providers’ knowledge of AEFI reporting. This study provides important insights into understanding challenges with COVID-19 AEFI surveillance and informs future preparedness efforts in Uzbekistan and neighboring countries with a similar context.

Our findings of low reporting of AEFI are consistent with those of published studies (15, 16). Healthcare providers were not able to follow up with patients to actively assess AEFI. Only patients with severe AEFI would have returned to the healthcare facility to report AEFI to providers. Nevertheless, the AEFI incidence for COVID-19 vaccines in our study was within the range of AEFI reported by countries, ranging from 0.5% AEFI in Canada (17) to 29% AEFI reported in Jordan (18) and 35.9% AEFI reported in Australia (15). The incidence of serious AEFI in the Khojaly district, Uzbekistan is 3 per 100,000 vaccine doses administered, which is lower than that reported in the Western Pacific region, where the prevalence of serious AEFI is 5.6 per 100,000 (19). However, due to gaps in AEFI registry and lack of documentation of these serious AEFI in outpatient medical records, there is a possibility that they were recorded erroneously. The highest percentage of AEFI in our study was attributed to entry errors, in contrast to other countries, which report primarily “main adverse events of special interest” (20). We identified that 75% of reported AEFI were entry errors. These errors were due to two main reasons: (1) providers recorded second and third dose vaccine refusals as an AEFI; and (2) the registry did not have an option for entering more than one vaccine name for patients who had received multiple doses with different vaccines. The gaps identified in the AEFI system data are consistent with what healthcare providers reported in our survey, which indicated that they do not fully understand the procedures for active detection, registration, and reporting of AEFI.

A lack of knowledge about how to report AEFI among providers is not unique to Uzbekistan. A study from Nepal reported that 34.9% of providers were unaware of where and how to report AEFI (21). Likewise, a study from Ghana highlighted the structural barriers of the surveillance system and behavioral factors that influence providers’ decisions to report AEFI (19). Our findings highlight the importance of regularly training healthcare workers in the detection and reporting of AEFI, especially when new vaccines are rolled out.

Our evaluation also revealed important gaps in the national COVID-19 vaccination registry, which limits data use. During the rapid rollout of the registry amid an emergency response to the COVID-19 pandemic, data use at the facility level was not a priority. Upgrades and enhancements to the national electronic registry can be made with input from healthcare facilities and district health departments to ensure that the data being entered are being used to make decisions at the local level.

This study has several limitations. First, the use of a small sample of healthcare workers limits the generalizability of the findings and is not representative of all healthcare workers in the district. Second, the results of this study cannot be extrapolated to other districts of Uzbekistan, which limits the broader application of the conclusions. However, observational studies in a single setting still provide valuable insights when they highlight system-level challenges that are relevant across similar healthcare contexts. A collection of small observational studies provides a more accurate and equitable evidence base for action in global health. Last, individual-level AEFI paper records were never created for use at the healthcare facility; it is not possible to directly compare healthcare facility data to national data in the electronic registry, and it limits the ability to assess data quality. This may affect the accuracy of our assessment of the completeness and consistency of reporting across systems.

Despite these limitations, a key strength of our study is the finding of a low rate of AEFI following COVID-19 vaccination in Khojaly, suggesting that vaccines used during the pandemic were relatively safe. Additional strengths include the use of a well-established surveillance system evaluation approach, data abstracted from multiple sources to assess and cross-validate AEFI, and the collection of survey data from primary care providers.

Our study focused on the AEFI system associated with COVID-19 vaccination. However, the issues identified, such as data entry errors, incomplete understanding of recording and reporting procedures, and insufficient knowledge about AEFI among healthcare workers, may reflect broader systemic weaknesses in pharmacovigilance and AEFI surveillance in general. The lack of an integrated electronic AEFI recording system that covers all types of vaccines limits the ability to fully analyze the entire pharmacovigilance system in the country. Additional studies may help determine whether the identified gaps are unique to COVID-19 vaccines or represent general weaknesses in the AEFI detection, recording, and reporting system in Uzbekistan.

## Conclusion

Our findings demonstrate that the AEFI system in Uzbekistan was able to capture and report on AEFI following immunization with COVID-19 vaccines. However, we found that errors in reporting likely occurred due to a lack of awareness among primary care providers about the mechanisms for collecting, recording, and reporting AEFI cases. Additional training for healthcare workers at the AEFI, simplification of the data, and making the data more usable may help strengthen the AEFI surveillance system in Uzbekistan.

## Data Availability

The data analyzed in this study is subject to the following licenses/restrictions: data might be made available for authorized researchers after application to the Ministry of Health of the Republic of Uzbekistan. The authors confirm that the manuscript is an honest, accurate, and transparent account of the investigation being reported; and that no important aspects of the study have been omitted. Requests to access these datasets should be directed to Botirjon Kurbanov, botirjon.kurbanov@ssv.uz.
