# Supplementary material for: COVID-19 vaccination pharmacovigilance in Khojaly district, Uzbekistan: an epidemiological evaluation
Source: Front Public Health. 2025 Jul 2;13:1520821. doi: 10.3389/fpubh.2025.1520821 (PMC12263602; doi:10.3389/fpubh.2025.1520821)
Supplement: Supplementary file 1 [file Data_Sheet_1.docx]

**COVID-19 Vaccination Pharmacovigilance in Khojaly District, Uzbekistan: An Epidemiological Evaluation**

**Appendix I: Анкета для собеседования / Questionnaire**

**Фамилия / Surname**

_________________

**Имя / Name**

_________________

**Возраст / Age**

_________________

**Пол / Sex**

*Мужской / Male*

*Женской / Female*

*Другой / Other*

**Поликлиника (Название или номер) / Outpatient clinic (Name or number)**

_____________________

**Телефон / Phone**

_____________________

**Должность / Position**

_____________________

**Стаж работы (года) / Work experience (years)**

____________________

**Согласны ли вы провести с вами опросник? /**

**Do you agree to participate in the survey?**

*Да / Yes*

*Нет / No*

**Согласно ли вы публиковать ответы опросника? /**

**Do you agree with answers to the survey to be published?**

*Да / Yes*

*Нет / No*

**Вы слышали по проявление после иммунизации (ПППИ)? /**

**Did you hear about adverse events following immunization (AEFI)?**

*Да / Yes*

*Нет / No*

*Не хочу отвечать / Do not want to answer*

**Вы регистрируете проявление после иммунизации (ПППИ)? /**

**Do you register adverse events following immunization (AEFI)?**

*Да / Yes*

*Нет / No*

*Не хочу отвечать / Do not want to answer*

**Как вы регистрируете? How do you register?**

___________________________

**Куда вы заносите? Where do you register?**

_______________________________

**Вы знаете как выглядит форма или бланк для регистрация ПППИ ? /**

**Do you know how adverse events following immunization (AEFI) registration form or blank looks like?**

*Да / Yes*

*Нет / No*

*Не хочу отвечать / Do not want to answer*

**Знаете какие виды есть ПППИ? /**

**What types of AEFI do you know about?**

*Да / Yes*

*Нет / No*

*Не хочу отвечать / Do not want to answer*

**Вы можете разделить легкий и тяжелый форма ПППИ? /**

**Can you distinguish between minor and severe forms of AEFI?**

*Да / Yes*

*Нет / No*

*Не хочу отвечать / Do not want to answer*

**Сколько раз вы регистрировали проявление после иммунизации (ПППИ)? /**

**How many times did you register adverse events following immunization (AEFI)?**

**_____________________**

**По тяжести какой вид ПППИ ( Легкий степен)/ What type of AEFI (minor)**

________________________

**По тяжести какой вид ПППИ. (Тяжелый степен)/ What type of AEFI (severe)**

________________________

**Сколько времени нужно чтобы для регистрации одного случая? /**

**How much time do you need to register one case?**

*5 минут / 5 minutes*

*10 минут / 10 minutes*

*Прочее / Other*

*Не знаю / Do not know*

**Если прочее указать (минут)/ If other how many (minutes)**

________________

**Знаете, кто может поставить диагноз проявление после иммунизации (ПППИ)? /**

**Do you know who can diagnose adverse events following immunization (AEFI)?**

_________________

**Если да кто? / If yes, who?**

________________

**Как вы думаете какие трудности есть регистрация проявление после иммунизации (ПППИ)? /**

**What do you think are the challenges when registering adverse events following immunization (AEFI)?**

____________________

**Если обнаружили ПППИ кому сообщите? /**

**Who do you report to when AEFI occurs?**

____________________

**Отчеты по вакцинации и ПППИ когда и кому сдаёте ? /**

**Who and when do you report AEFI?**

___________________

**Вы проверяйте еженедельные отчеты и считаете вы их важными для принятия решений? /**

**Do you check weekly reports and do you consider them important in making decisions?**

___________________

**Как вы думаете как мы улучшаем качество выявления ПППИ? /**

**How do you think we improve the quality of AEFI detection?**

____________________

**Проводите ли вы разъяснительную работу среди населения? /**

**Are you conducting outreach work among the population?**

*Да / Yes*

*Нет / No*

*Не хочу отвечать / Do not want to answer*
